# A nonlinear approach to identify pathological change of thyroid nodules based on statistical analysis of ultrasound RF signals

**DOI:** 10.1038/s41598-017-17196-2

**Published:** 2017-12-05

**Authors:** Huan Xu, Chunrui Liu, Ping Yang, Juan Tu, Bin Yang, Dong Zhang

**Affiliations:** 1 Key Laboratory of Modern Acoustics (MOE), Department of Physics, Collaborative Innovation Center of Advanced Microstructure, Nanjing University, Nanjing, 210093 China; 2 National Institute of Metrology, Beijing, 100029 China; 3 Department of Ultrasound, Jinling Hospital, Medical School of Nanjing University, Nanjing, 210016 China; 4 The State Key Laboratory of Acoustics, Chinese Academy of Science, Beijing, 10080 China

## Abstract

In order to reassure the majority of patients with benign nodules from unnecessary needle biopsy, there is an increasing clinical requirement to identify benign and malignant thyroid nodules during ultrasound diagnosis. A nonlinear approach based on statistical analysis of ultrasound radio-frequency (RF) signals was developed for differential diagnosing the thyroid nodules to improve the diagnostic accuracy. Data from 44 patients with solitary thyroid nodules were collected, following with the ultrasound-guided fine needle aspiration (FNA) as the ground truth. The relative *P*-value (*rP*-value) was estimated to quantify the pathophysiologic changes by comparing the region of interest (ROI) with the no pathological change part in the thyroid gland using only one frame of raw RF data. The malignant nodules were distinguished from benign ones with high accuracy and high credibility (sensitivity = 100%, specificity = 80%). Suspicious nodules (*rP*-value < 0.5) could be picked out for FNA with no additional instruments. This method shows promising in differentiating malignant from benign thyroid nodules, subsequently avoiding unnecessary biopsies.

## Introduction

Thyroid nodules become very common in the population in recent years. Although the majority of nodules are benign^1,2^, the number of malignant thyroid nodules rises rapidly^3,4^. Papillary thyroid microcarcinoma which is the most prevalent thyroid cancer is reported to be high^3–6^. The B-mode ultrasonography is widely employed to detect and classify abnormalities of the thyroid gland, taking the advantage of non-invasion, real-time, convenience and high sensitivity. This technology can diagnose abnormal tissues by detecting their changes in the acoustic properties through identifying not only the shapes of important structures but also the changes in their texture. A nodule detected by B-mode imaging can be characterized as hyper-echoic, iso-echoic, or hypo-echoic. However, the complex inner echogenicities of thyroid nodules and multiple surrounding tissues make the diagnosis difficult^7^. Although several ultrasound features, including hypo-echogenicity, blurred or spiculate margins, intranodular vascularity, and insistence of calcifications have been found to be associated with thyroid cancer, it is hard for clinicians to distinguish the malignant nodules from benign ones by these features or their combination^8,9^. Moreover, the diagnosis is constrained by the observations of the sonographer, and it is relatively subjective to draw conclusions by combining so many ultrasound indicators in surgeons’ or endocrinologists’ medical knowledge. Therefore, in spite of those ultrasonographic findings that can help screening malignancy, ultrasound-guided fine needle aspiration (FNA) remains the gold-standard for distinguishing benign and malignant thyroid nodules because of its high specificity and sensitivity^10,11^. However, FNA is relatively invasive, costly and uncomfortable for patients.

To avoid unnecessary needle biopsy, it is urgent to develop tools assisting the clinicians to diagnose the thyroid nodules. This auxiliary tool is aimed at releasing the most of patients who have benign nodules from FNA and providing prompt diagnosis for the patients with the malignant nodules. Ultrasound elastography has been developed for this clinical requirement^12,13^. On the basis of the principle that the malignant thyroid nodule is stiffer than benign or normal one, ultrasound elastography estimates tissue elasticity by measuring the degree of distortion of the ultrasound beam in response to a standardized external force. Although this technique have a good performance in the detection of malignancy in thyroid gland and other organs^12–14^, Rago *et al*. mentioned that this technique can’t be used on nodules less than 8 mm, containing >20% cystic, with coarse calcifications, or coalescent nodules^12^. Moreover, Vidal-Casariego *et al*. pointed out that elastography lacked accuracy for the diagnosis of malignant nodules in low-risk population^15^. Recently, people show great interests on employing artificial intelligence to identify pathological changes^16,17^. The computer-aided diagnose (CAD) systems based on extracting features from the B-mode images are also developed to classify tumors^18,19^. Different tissues with gray-level intensity have markedly different texture in the B-mode image. This technology has been applied in diagnosing some organs, such as breasts, livers and prostate. However, it was rarely applied to identify thyroid nodules, because of its complex and various textures. The B-mode image is obtained from the amplitude of the backscattered echo signals only, omitting other information of the target area during the image processing. Consequently, the raw radio-frequency (RF) data are more informative than the processed B-mode image for the characterization of tissues^20–22^. Statistical analysis was adopted to describe the pathological characteristics of tissues by building the distribution functions of the envelope of backscattered RF signals^23^. Several distributions, including Rayleigh distribution, K-distribution, Nakagami distribution, gamma distribution or their compound, were developed to achieve better fits to the envelope of the RF signals^24^.

Lots of researches showed that the acoustic nonlinearity parameter B/A can reflect the structural features and the pathological change of the tissue^25–27^, and determine the 2^nd^ harmonics scattered by tissues^25–27^. Thus, the tissue harmonic imaging that measures B/A in the tissue has the potential to detect the abnormality in biological tissues^28–30^. Taking the benefits of improved lateral resolution, reduced side lobe artifact, and increased signal-to-noise^28–30^, the information carried by 2^nd^ harmonics will have higher quality than fundamental signal. For instance, Tranquart *et al* ^28^. compared the images collected for different organs (e.g., kidney and gallbladder) by using both 2^nd^ harmonic imaging and conventional imaging. They concluded that, comparing with conventional sonography, the better conspicuity of septas, calcifications, or nodules could be identified in the 2^nd^ images of kidney, and clearer differentiation of gallbladder sludge from artefacts as well as stone detection could be realized in the 2^nd^ images of gallbladder. The studies for the pancreas also showed that harmonic sonography was significantly better than conventional sonography at 2.5 and 4 MHz for penetration, detail and overall image quality, so that the detection of carcinomas of the pancreatic head could be easier^31^. Therefore, these researches suggested that the 2^nd^ harmonics were more informative than fundamentals. One can also expect that 2^nd^ harmonic signals extracted from the RF echo signals of tissue harmonic imaging to give more information than the fundamental signals collected by conventional ultrasonography. This work aims to develop a nonlinear approach to identify the malignant thyroid nodules from benign ones by comparing the statistical property (*P*-value) of region of interest (ROI) with that of the normal part in the thyroid gland within the same frame of RF signals. The relative *P*-value (*rP*-value) which presents the distributional abnormity of ROI was calculated. Two-tailed Mann-Whitney *U* test was performed to determine whether the *rP*-values for the benign and malignant nodules were significantly different. Furthermore, a threshold for suspicious nodules was set for further FNA.

## Results

### Characteristics of thyroid nodules

**Figure 1:**
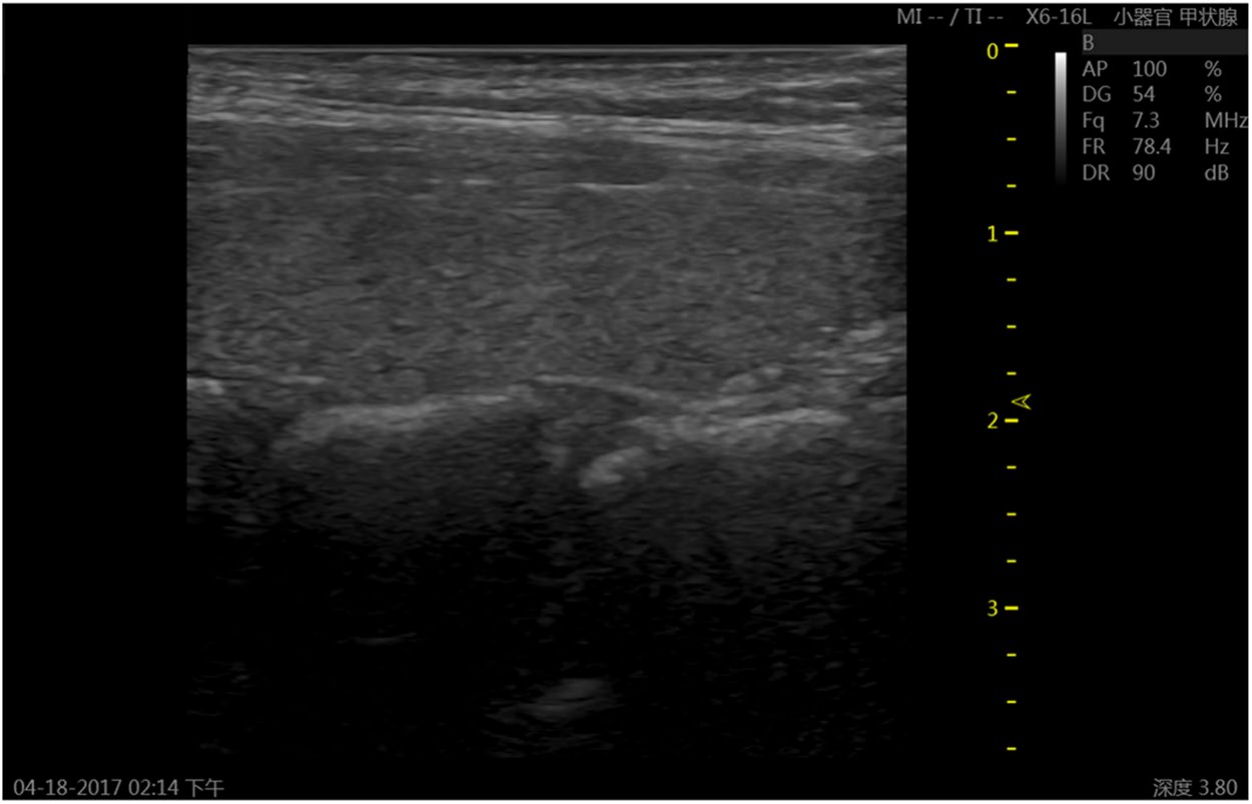
A sample image of normal thyroid gland.

**Figure 2:**
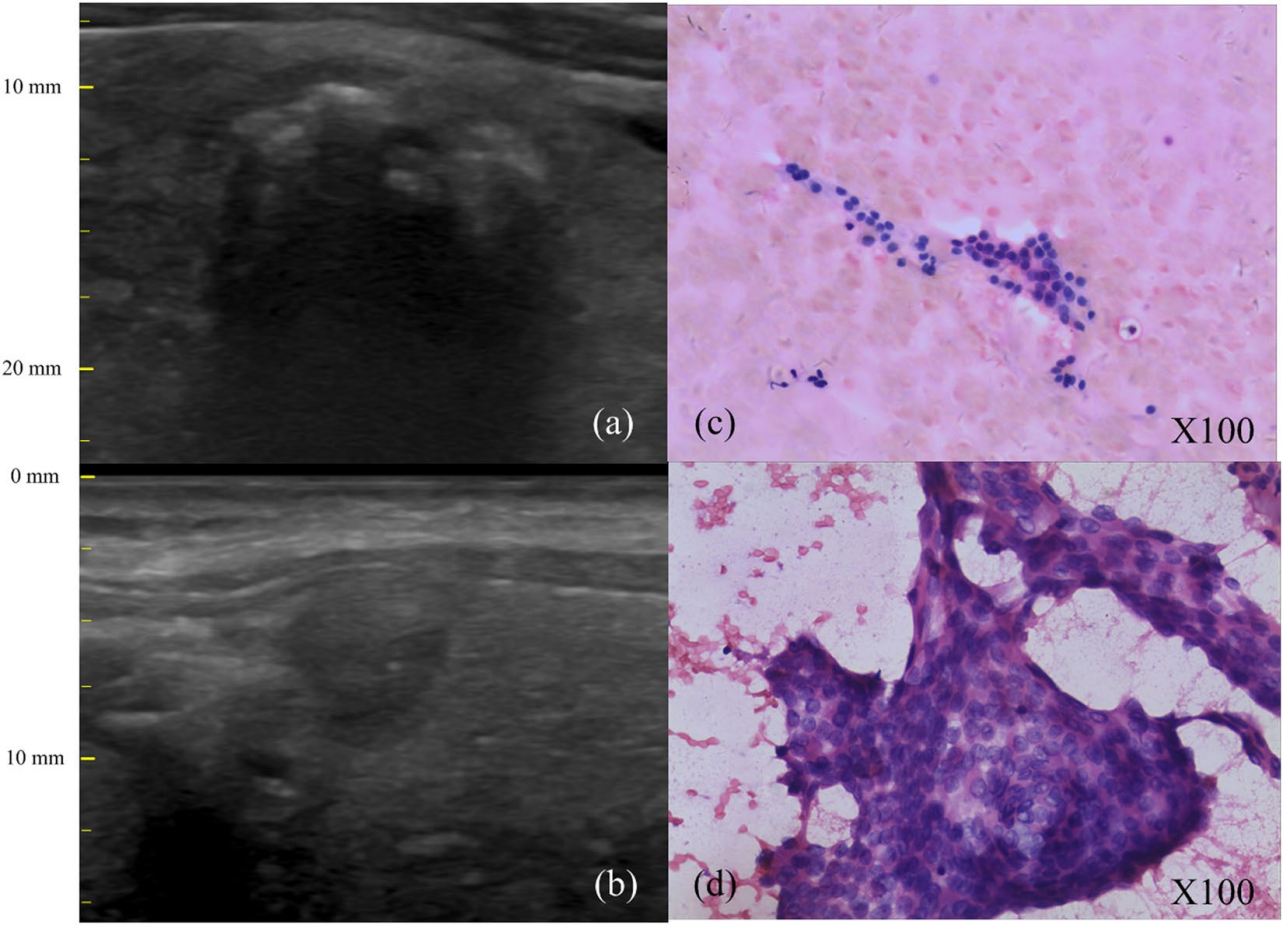
The B-mode ultrasonography and FNAs of thyroid nodules: (**a**) the B-mode image of one benign thyroid nodule and (**c**) its corresponding photomicrograph on FNA smears; (**b**) the B-mode image of one malignant thyroid nodule and (**d**) its corresponding photomicrograph on FNA smears.

Figure 1 shows the longitudinal B-mode image lobe of a normal thyroid gland, oval. The thyroid gland was typically located with regular well-defined margins and homogeneous isoechoic structure. The capsule was uniform and continuous on all extent. Pathological change of the thyroid gland can be detected by B-mode ultrasonography, as its echogenicity changed. Clinicians identified the thyroid nodules through the boundary and inner texture of ROI in the B-mode images. Figure 2 showed the B-mode images and the corresponding FNAs of a benign thyroid nodule and a malignant thyroid nodule, respectively.

Benign thyroid nodule: A case of nodular goiter with hyperechogenicity, coarse calcifications and acoustic shadow was observed in the B-mode image (Fig. 2a). The FNA smears of this nodule showed that the thyroid follicular cells were evenly spaced and had a small and uniform nuclear size (Fig. 2c). The FNA result suggested that it was a benign follicular nodule.

Malignant thyroid nodule: The B-mode image (Fig. 2b) showed inhomogeneous hypoechogenicity structure and indistinct margins. Additionally, the corresponding FNA smears showed a cellular aspirate with numerous abnormal follicular cells containing enlarged hyperchromatic nuclei, nuclear pseudoinclusions (Fig. 2d). A typical papillary thyroid carcinoma was observed.

Through the observations of both the B-mode ultrasonography and the FNA, one can suppose that the texture of the malignant nodules changed significantly and distributed more disorderly than benign ones. In the current study, twenty-four malignant thyroid nodules and twenty benign nodules were collected. The characteristics of all these subjects, including the nodule size measured by the B-mode ultrasonography and the FNA results, were given in Table 1.

**Table 1:** Summary of the study subjects.

Subjects	Nodule size (mm × mm)	FNA results	Subjects	Nodule size (mm × mm)	FNA results
1	6 × 6	malignant	23	7 × 8	malignant
2	8 × 9	malignant	24	4 × 3	benign
3	17 × 22	benign	25	10 × 6	malignant
4	8 × 12	malignant	26	5 × 6	malignant
5	9 × 8	malignant	27	8 × 7	malignant
6	9 × 5	benign	28	19 × 7	benign
7	13 × 11	malignant	29	10 × 8	malignant
8	6.3 × 3.3	benign	30	18 × 11	malignant
9	16 × 10	malignant	31	10 × 6	benign
10	8 × 6	benign	32	5 × 6	malignant
11	6 × 5	malignant	33	8.5 × 5	benign
12	9 × 7	malignant	34	17 × 11	benign
13	13 × 13	benign	35	24 × 17	benign
14	15 × 10	malignant	36	10 × 7	benign
15	10 × 6	malignant	37	27 × 16	malignant
16	12 × 15	benign	38	13 × 22	benign
17	13 × 10	benign	39	20 × 15	malignant
18	21 × 13	benign	40	4.6 × 4.3	benign
19	14 × 15	benign	41	8 × 10	malignant
20	33 × 19	malignant	42	9 × 15	malignant
21	5 × 2	malignant	43	9 × 8.7	benign
22	11 × 9	malignant	44	7 × 5	benign

### *P*-value method

**Figure 3:**
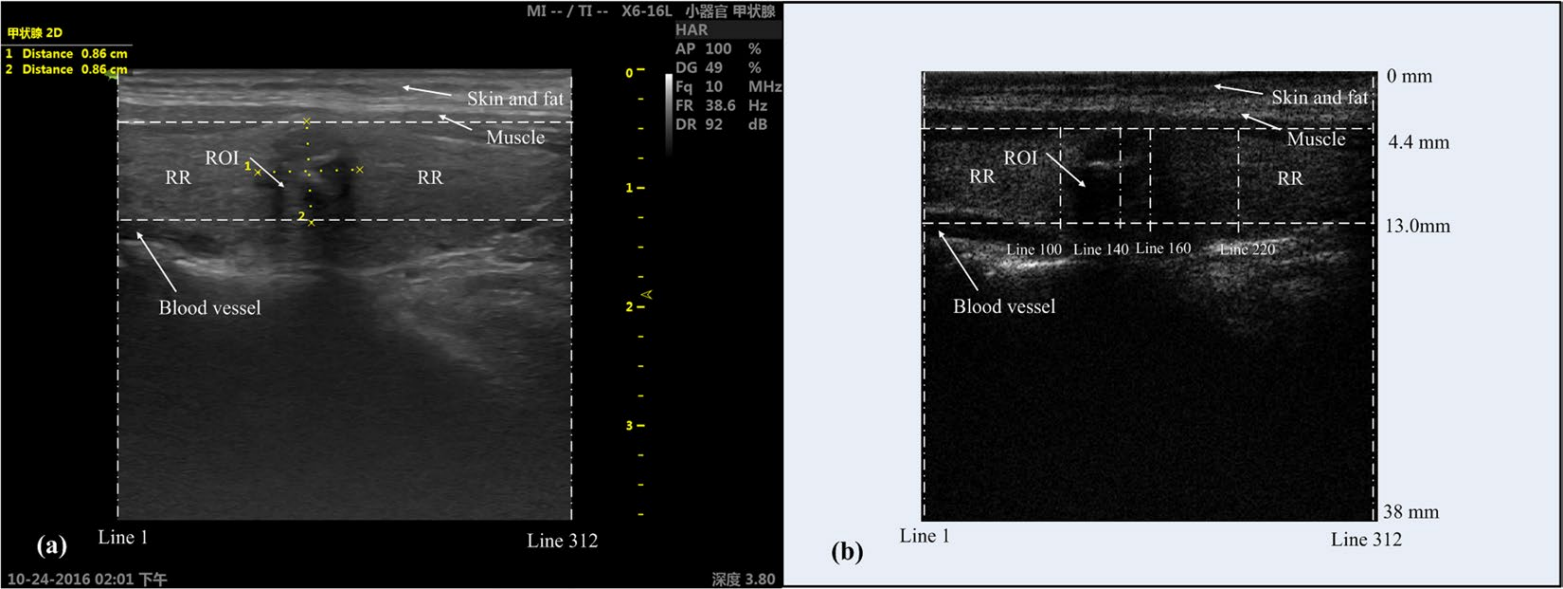
A typical thyroid nodule illustrated in: (**a**) the B-mode image; (**b**) the RF image frame.

This method was performed by assuming that the amplitude of the echo signals along the scanning lines in ROI were distributed differently with those in reference region (RR) which has no pathological change. Figure 3 illustrates a typical thyroid nodule in both the B-mode image (a) and corresponding RF image frame (b). In general, the RF image frame (e.g., Fig. 3b) was constructed through the following processes: 1) acquiring raw RF data using the diagnostic ultrasound system; 2) extracting 2^nd^ harmonics from raw RF data by applying a band-pass filter (7.5 MHz~12.5 MHz); 3) deriving the envolpes of filtered 2^nd^ harmonic signals by Hilbert transform; and 4) transferring 2^nd^ harmonic amplitudes to gray scale values and constructing the gray-scale RF image.

**Figure 4:**
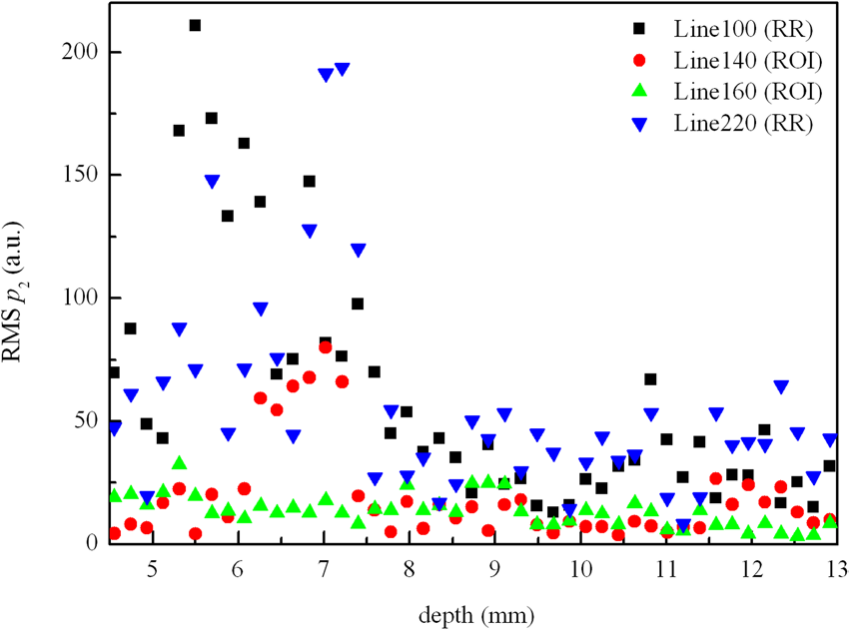
The RMSs along four scanning lines.

**Figure 5:**
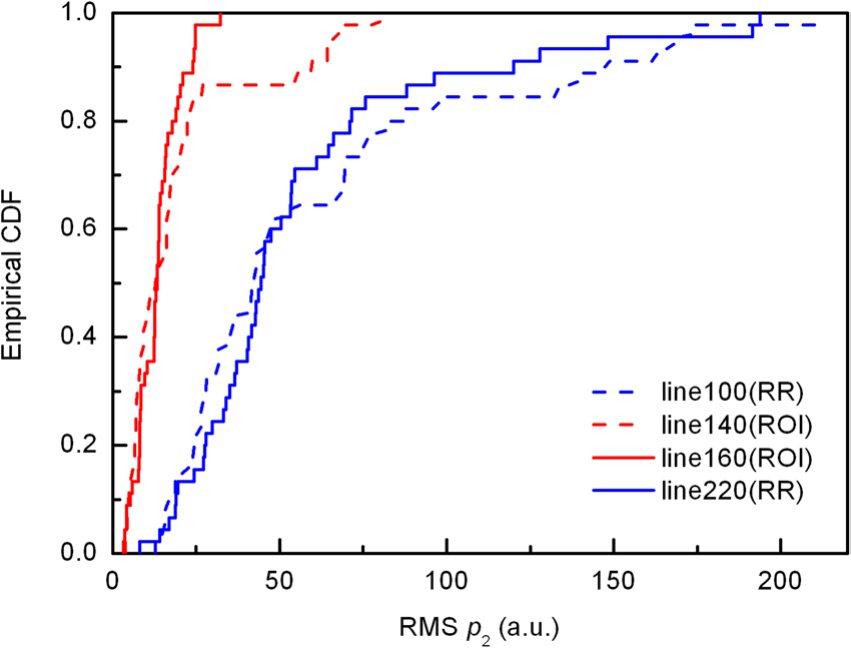
The empirical CDF of four lines in different regions in one frame of RF data for the thyroid nodule in Fig. 3.

To show the distribution difference between the two regions, four different scanning lines in one frame of RF data of sample No. 2 were selected, viz., 140^th^ line and 160^th^ line in ROI, 100^th^ line in the left RR, and 220^th^ line in the right RR. The regions and lines are illustrated in the image of the RF frame (Fig. 3b). The total scanning depth of the RF frame shown in Fig. 3b is 38 mm, and the depth of selected region is between 4.4 mm to 13 mm. The nodule position in the RF frame (Fig. 3b) might be slightly different with that in the B-mode image (Fig. 3a) due to slight movement of the sonographer or the patient. Figure 4 illustrates the RMS amplitudes calculated for the 2^nd^ harmonic signals along individual lines in selected regions (viz., Lines 100, 140, 160 and 220). The RMS along two lines in ROI is generally lower than those in RR, indicating obvious hypoechogenicity structure of malignant nodules. Then, the empirical cumulative distribution functions (CDF) of the RMSs along individual lines were calculated to illustrate the distribution difference between ROI and RR as shown in Fig. 5. If the two groups distributed similarly, their CDFs should be close. However, one can observe in Fig. 5 that, the CDFs of the lines in RR are near to each other, while far away from the CDFs of the lines in ROI.

**Figure 6:**
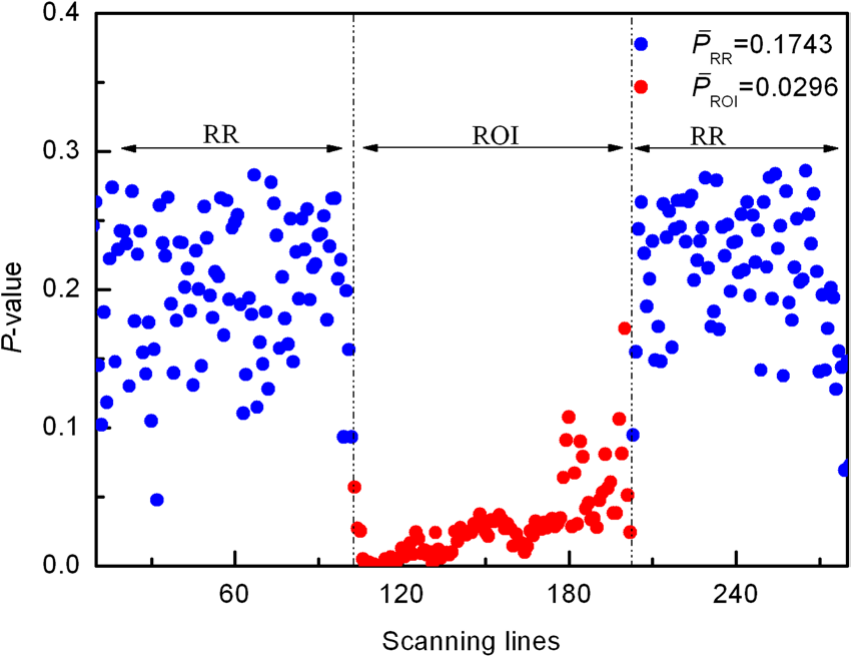
The *P*-values of the scanning lines. The relative *P*-value for this sample can be deduced from Eq. (1): *rP*-value = 0.1699.

**Figure 7:**
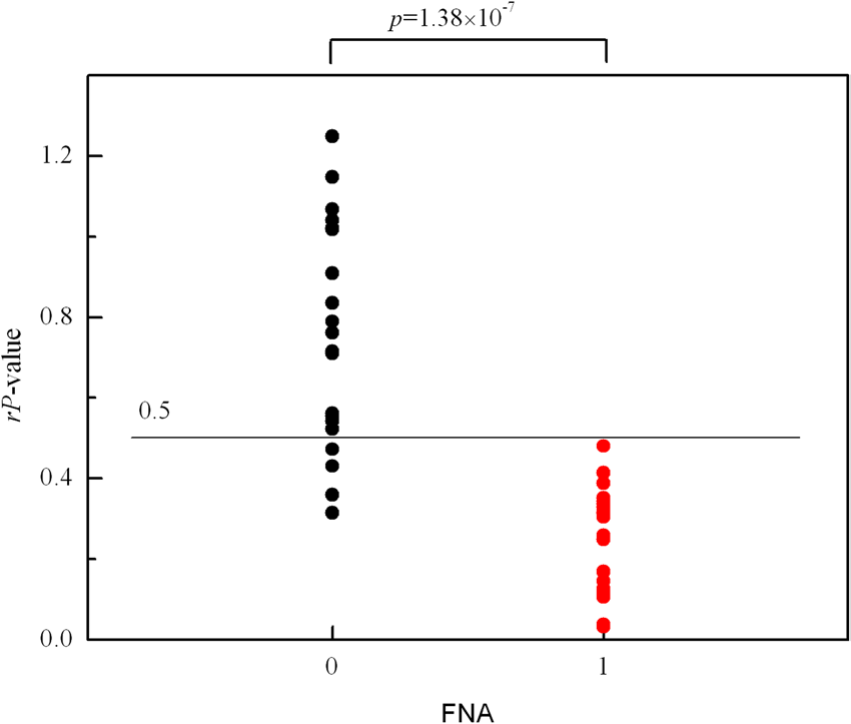
The distribution of *rP*-values for 44 thyroid nodules: benign nodules ‘0’ and malignant nodule ‘1’. A cut-off value for *rP*-value of 0.5 gave a sensitivity of 100% and specificity of 80%. Horizontal line showed the cut-off point at *rP*-value = 0.5.

In order to quantify the abnormality of this thyroid nodule, for a typical line in the 2^nd^ harmonic RF image, its RMS was calculated and compared with individual RMSs obtained for the lines in RR one by one, based on two-tailed two sample Kolmogorov–Smirnov test (K-S test). Then, by averaging the *P-*values given by the K-S test, an effective *P-*value could be estimated for this typical line, which could be used to evaluate its difference from the lines in RR. Eventually, greater changes could be identified by smaller effective *P-*value. The *P-*values for all the scanning lines in the RF frame are shown in Fig. 6. It was observed that the *P-*values in ROI were significantly smaller than those in RR. The arithmetic means for the two regions were: $\overline{{p}_{RR}}=0.1743,\overline{{p}_{ROI}}=0.0296$. Then the relative *P*-value (*rP*-value) between the two regions can be evaluated to present the pathological change of ROI. The *rP*-values for all the thyroid nodules were calculated without considering the scanning direction (Fig. 7). Obviously, the *rP*-values of malignant nodules were smaller than those of benign nodules. The results proved that malignant thyroid nodules distributed more differently from the normal thyroid gland than benign nodules. Meanwhile, significant differences (*p* = 1.38 × 10^−7^, two-tailed) were observed in *rP*-values between the 20 benign nodules and 24 the malignant thyroid nodules by Mann-Whitney *U* test (alpha level: α = 0.01). An *rP*-value threshold, below which the nodules should be suspicious, was set to detect all malignant nodules for further diagnosis. As shown in Fig. 7, by adopting an *rP*-value threshold of 0.5, all the malignant nodules could be detected and 80% benign nodule could be released from unnecessary FNA (sensitivity = 100%, specificity = 80%).

## Discussion

A novel nonlinear approach for differentiating the malignant thyroid nodules from benign ones was presented in the current work. The *P*-value method extracted nonlinear features from the 2^nd^ harmonic echo signals which contained more information on pathological change of the tissue than the fundamental signals. It was based upon the principle that the RMSs of 2^nd^ harmonic echo signals in malignant thyroid nodules distributed more differently than those in benign nodules. The *rP*-value which represented the statistical difference of lesions was estimated by comparing the ROI with the normal part in thyroid gland. This method was easy to operate and required only raw RF data with no need for additional instrumentation. Moreover, it used only one frame of RF data, avoiding the error induced by the displacement of the patient or clinician. Additionally, the uncertainty induced by the noise and other tissues was minimized by selecting regions and averaging the *P*-values in the regions. As shown in Fig. 3, the RR which was supposed to be the normal part in thyroid gland unavoidably contained parts of other tissues, for example skin, fat, muscle and blood vessel. Even the weak coupling between the probe and the skin may cause the distribution in those areas deviating from others, the weak coupling varied in different frames, and it was impossible to exactly identify its coverage area. To standardize our processing procedure, the boundary was not excluded and averaging algorithm was done to minimize this influence. If a nodule was statistically more similar to the normal part than the error sources included in RR, the *rP*-value for this nodule may be larger than 1 (Fig. 7). On the other hand, when these errors included in RR played more important role than the normal part, it will lead to false diagnosis. Benign nodules may be classified as suspicious nodules, if lower *rP*-value was derived. Obviously, the *P*-value method can’t be applied to large nodules which overlapped all the lines in one frame, since there was no normal part left in the select region.

In the present work, the position of the thyroid nodule (ROI) was determined by an experienced sonographer. Although it was difficult to exactly differentiate whether the scanning line around the nodule boundary belonged RR or ROI, the robustness of the current algorithm still could be verified by the following analyses. For the thyroid illustrated in Fig. 3, the r*P*-values of all the 20 frames were calculated respectively. The results listed in Table 2 give a mean value of 0.1671 with a standard deviation as small as 0.0189, which suggests that the current algorithm should be robust enough.

**Table 2:** The calculated *rP*-values from 20 RF frames.

Frame No.	*rP*-value	Frame No.	*rP*-value
1	0.1751	11	0.1429
2	0.1398	12	0.1803
3	0.2081	13	0.1744
4	0.1763	14	0.1783
5	0.1657	15	0.1457
6	0.1417	16	0.1761
7	0.1597	17	0.1785
8	0.1420	18	0.1627
9	0.1619	19	0.1699
10	0.2015	20	0.1618

It should pointed out that, with referring to the common protocol adopted by other researchers^18,19,22^, we just set the ROI to be the region of nodule identified by experienced sonographer and the RR to be surrounding tissue area. Moreover, the difference between ROI and RR might be blurred due to the low signal-to-noise ratio, and the signal-to-noise ratio might be affected by the individual difference of patients. Therefore, more efforts certainly need to be made in our future work to develop more powerful adaptive algorithms to automatically select ROI and RR with greater accuracy and objectivity, and collected a large amount of data to determine the optimized region of signal-to-noise ratio best suitable for the current method.

Although this method showed some limitations, it can distinguish the thyroid nodules which were hard to distinguish by other methods. As mentioned by Dighe *et al*.^32^, differential diagnosing small thyroid nodules (<10 mm) can be technically challenging even by FNA. The ultrasound features suggesting malignancy may not be seen in some small nodules due to the difficulty in assessing the internal architecture. Furthermore, they evaluated the efficacy of distinguish small thyroid nodules by ultrasound elastography, and a sensitivity of 100% and a specificity of 60% in detecting papillary microcarcinomas were achieved by using an elasticity contrast index cut-off value of 3.6. Rago *et al*. also mentioned that the ultrasound elastography didn’t show reliable results on classifying nodules less than 8 mm^12^. In the current study, the nodule size in the data set ranged from 5 × 2 mm^2^ to 27 × 16 mm^2^, and over half of them were small nodules. High sensitivity and specification were achieved by using the *P*-value method, that can hence lead to an increase in confidence and accuracy in deciding whether a nodule is suspicious for malignancy or not.

Several models have been developed to statistically characterize tissues by the ultrasound RF signals, for example Rayleigh distribution, K-distribution and Nakagami Distribution^23,24^. These distributions have shown good performances on specific conditions, e.g, the Rayleigh distribution can fit the data well when the speckle signal-to-noise ratio is 1.91^24^. Although these models have applied to detect some abnormal tissue^23^, they are rarely applied in classifying thyroid nodules. Comparing with previous methods, the current *P*-value method can extract new feature, r*P*-value, which estimated the lesion abnormality. In addition, the data were collected under daily diagnosis without any special specifications, which makes it more practicable in clinics. This newly developed technique may be utilized in distinguishing abnormality of other organs without considering the backscattered echo signals fulfill what kind of distributions.

It should be mentioned that the *P*-value method can’t be applied to large nodules that overlapped all the lines in one frame. Other features of large ones can be applied for the classification, e.g., the elasticity contrast index measured by ultrasound elastography, and the fractal dimension evaluating the random texture of lesions^19^. As mentioned in the paper, the ultrasound elastography shows poor performance on small nodules. However, the currently proposed method can be used as an effective tool to identify small ones. To make accurate classification on all nodules, a system based on learning machine might be developed to combine all the features.

In conclusion, a nonlinear approach based on statistical analysis of ultrasound RF signals was developed for the thyroid nodules diagnosis. This method compared the nodules identified by ultrasonography with the normal part in the thyroid gland, by using only one frame of RF raw data. A new parameter, *rP*-value, was proposed to describe the abnormality of thyroid nodules. The outcome of this method agreed well with the results of FNA. By using the current method, suspicious nodules (*rP*-value < 0.5), which was highly suggested to be malignant, could be distinguished earlier before FNA. This method was easy to operate and needed no additional equipment. Therefore, it can be developed as an auxiliary tool for ultrasound diagnose of thyroid disease, helping with picking out suspicious nodules for FNA so as to avoid unnecessary FNA for benign ones.

## Materials and Methods

### Data acquisition

The B-mode ultrasonography were performed by a commercially available Vinno70 color Doppler US system (VINNO Technology (Suzhou) Co., Ltd.) with an ×6-16 L broadband probe (6~18 MHz). The research platform provided by the manufacturer enabled acquisition of beamformed RF signals in real time. The device was set on tissue harmonic imaging mode with the 2^nd^ harmonic frequency of probe fixed at 10 MHz, which means the fundamental frequency was 5 MHz. Other parameters, such as the mechanical index (MI), depth, thermal index of soft tissue (TIS) and total gain, were decided by the clinical need. The RF data was sampled at 50 MHz and 312 scan lines were acquired for each frame. In one single scan, twenty frames were saved for each patient within one second, according to the default setting of the machine. Only one frame was randomly selected for the *P*-value analysis. Following the B-mode diagnosis, the FNA was taken as the ground truth. It was performed using a 24 G needle under ultrasonographic guidance by an interventional radiologist. After determining the position of the nodule with ultrasonic guidance, several samples inside of the nodule within the ultrasound scanning plane were obtained using the needle. Four or five biopsy specimens were fixed in 95% ethyl alcohol and then hematoxylin eosin (HE) staining. All slides were reviewed and interpreted by three practiced cytotechnologists referring to the Bethesda system for reporting thyroid cytopathology. Histological results were used in lieu of cytology if the nodule underwent surgical resection. A final pathological diagnosis of benign or malignant was assigned to a thyroid nodule if it had a benign or malignant cytology (or histology if available). Fifty three thyroid nodules with suspicious ultrasound features from 53 patients were collected by from October 24^th^ to November 17^th^ in 2016. Since the reference region was necessary to compare with ROI, the data of 9 nodules were excluded in the final analysis as the tumor size is too large to cover all lines in the RF frame.

### *P-*value method

The acoustic nonlinearity parameter B/A reflects the acoustical properties of the mediums. B/A is defined as $B/A=2{\rho }_{0}{c}_{0}{(\partial c/\partial p)}_{0,s},$ where *ρ*_0_ and *c*_0_ are the density and sound speed of the medium, *p* is static pressure and *s* is entropy^25^. This equation indicates that B/A reflects the stress dependence of elastic parameters. This parameter is correlated to the pathological change of tissues^25–27^. The acoustic nonlinearity imaging which measures B/A in the tissue shows good performance on identifying different tissue structures and detecting the abnormality of tissues^25,27^. Since B/A determines the 2^nd^ harmonics *p*_2_, the abnormality of the ROI in the tissue can be calculated by comparing the 2^nd^ harmonics *p*_2_ in ROI with the ones in normal area. In current work, the ultrasound machine was tuned in the tissue harmonic imaging mode, the frequency of the 2^nd^ harmonic signal was set to be 10 MHz, and the RF data were collected at a sample rate of 50 MHz. Then the raw RF data were filtered by a bandpass FIR filter with bandwidth from 7.5 MHz to 12.5 MHz to remove noise superimposed in the RF frame. The RMSs of the 2^nd^ harmonic wave along each line were calculated for every ten points (about 0.13 mm length).

As shown in Fig. 3a, there are always some other tissues (e.g., blood vessel, muscle, skin and fat) surrounding the ROI, which might be irrelevant to the diagnosis of nodulus. Therefore, in order to save the computation time, a band between two horizontal dash lines in Fig. 3b is selected as the processing area following the principle that the upper and lower boundaries of ROI are completed covered. The ROI was defined by experienced sonographer in this study. The rest part in the selected area other than ROI was regarded as normal tissues without pathological changes and could be used as the reference region for ROI (viz., RR). Then, a group of RMSs of the 2^nd^ harmonic signals was calculated for individual lines in the processing area. To quantify the abnormality of the thyroid nodule, the RMSs of each line in the 2^nd^ harmonic RF image were compared with the RMSs obtained for individual lines in RR one by one, through two-tailed two sample K-S test. The two-tailed two-sample K-S test quantifies a distance between the empirical distribution functions of two samples, and returns asymptotic *P*-value which presents the similarity of the two distributions. The *P*-value is distributed between zero and one, “one” means the two samples are equal, and “zero” means they are totally different. Eventually, by averaging the *P-*values given by the K-S test, an effective *P-*value could be estimated for the typical line, which could be used to evaluate its difference from the lines in RR.

To standardize our algorithm and minimize the error induced by tissue variety and complexity, all 312 lines in the select region were divided into the two parts (ROI or RR) and the average *P*-value of each part was applied. The *rP*-value ${p}_{relative}$ which described the abnormality of ROI was calculated to make sure different RF data of nodules collected under different conditions comparable. It can be presented as equation (1),

1 $${p}_{relative}=\frac{\overline{{p}_{ROI}}}{\overline{{p}_{RR}}}$$

where $\overline{{p}_{ROI}}$ and $\overline{{p}_{RR}}$ are the averaged *P-*value for the ROI and RR, respectively. All the calculations were processed in Matlab program (Math Works, Natick, MA).

### Ethics

This study was reviewed and deemed exempt from written informed consent by the Institutional Review Board (IRB) of the Jinling Hospital at Medical School of Nanjing University. The patient records were anonymized and de-identified prior to analysis. It was approved by the IRB for analysis.
